# Nonoperative Management of Perforated Diverticulitis of the Duodenum: Report of Three Cases and Review of Literature

**DOI:** 10.1155/2021/6647470

**Published:** 2021-02-20

**Authors:** Rutger Franken, Martijn Möllers, Alexander de Mol van Otterloo, Julien Puylaert

**Affiliations:** ^1^Department of Surgery, Spaarne Gasthuis Hoofddorp, Spaarnepoort 1, 2134 TM Hoofddorp, Netherlands; ^2^Department of Surgery, Nij Smellinghe Drachten, Compagnonsplein 1, 9202 NN Drachten, Netherlands; ^3^Department of Surgery, Haaglanden Medical Centre Westeinde, Lijnbaan 32 2512 VA The Hague, Netherlands; ^4^Department of Radiology, Haaglanden Medical Centre Westeinde, Lijnbaan 32 2512 VA The Hague, Netherlands

## Abstract

Duodenal diverticula are relatively frequent but complications are uncommon. The mortality rate of perforated duodenal diverticulitis is high, and its management is controversial. We report three patients with a perforated duodenal diverticulitis who were successfully treated with conservative antibiotic therapy. The clinical presentation in all three patients was acute onset of pain in the upper abdomen. In all cases, ultrasound showed no abnormalities, but computed tomography revealed the correct diagnosis. All three were treated with broad-spectrum antibiotics and total parenteral nutrition. They recovered clinically and laboratory findings normalized. During follow-up visit, all patients were asymptomatic. This study contributes another three patients to the small number of successful conservatively treated cases of perforated duodenal diverticulitis described in literature. We suggest that in patients in good condition with no septic signs, conservative treatment with close clinical follow-up should be the treatment of choice.

## 1. Introduction

Duodenal diverticula are a relatively common anomaly with a frequency ranging from 5 to 22% in the entire population [1]. Exact data on the incidence of duodenal diverticulitis has not been described, and this pathologic entity seems relatively rare in patients presenting with acute abdominal pain. Among complications, perforation occurs rarely and is usually retroperitoneally. Perforation is the most threatening complication, and in literature, there is no consensus about the treatment of choice. When the diagnosis is unclear, an exploratory laparoscopy or laparotomy is performed and surgical resection is undertaken in some cases, with a mortality up to 20% [2]. The choice of procedure depends on location of the defect, the amount of tissue lost, and the amount of peritoneal contamination. CT scan in patients with acute abdominal pain has become a reliable diagnostic tool. It allows a noninvasive diagnosis of perforated duodenal diverticulitis [3–5]. In several reported cases, in which the diagnosis was made by CT scan, conservative management has been reported [6–13]. Since cases treated conservatively in literature are very scarce, percentages on complications and mortality are unknown. We describe another three patients with a perforated duodenal diverticulitis who were successfully treated by conservative management.

## 2. Case Reports

### 2.1. Case 1

**Figure 1:**
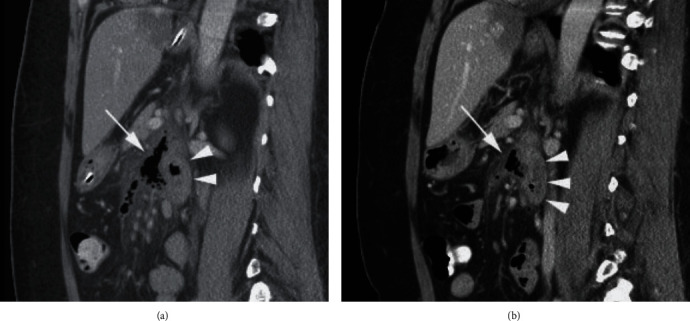
Retroperitoneally perforating duodenal diverticulitis. (a) Sagittal CT scan after intravenous and oral contrast. Ventrally from the duodenum (arrowheads), irregular air configurations are found at the location of the inflamed duodenal diverticulum (arrow). (b) After one week of conservative therapy, the air configurations are almost limited to the duodenal diverticulum itself (arrow). The duodenum (arrowheads) still shows mild wall thickening.

A 56-year-old adipose woman with no significant medical history presented with acute onset of sharp pain in the left upper abdomen. Her symptoms existed since the night prior to presentation. On physical examination, the patient was in mild discomfort with a body temperature of 39.4 degrees Celsius. Blood pressure and pulse rate were normal. The abdomen was not rigid, but there was direct tenderness on the left upper quadrant without muscle guarding. Blood analysis showed a mild leucocytosis (10.5∗10^3^ mm^3^ (4–10)) and normal c-reactive protein-level (<5 mg/L (<5)). Thoracic X-ray and abdominal ultrasound were normal. Abdominal computed tomography (CT) with intravenous contrast revealed a diverticulum located at the 4^th^ portion of the duodenum at the ligament of Treitz, with the suggestion of free air in the retroperitoneum (Figure 1(a)). The patient was admitted, restricted from oral intake, and placed under close observation with serial abdominal exams and laboratory evaluation. A nasogastric tube was applied for decompression. Intravenous broad-spectrum antibiotics were started directly after admission (metronidazole 3 dd 750 mg, cefuroxime 3 dd 750 mg, and gentamycin 1 dd 500 mg). Gentamycin dose was calculated by body weight. On the first day after admission, repeated blood analyses showed a rise in c-reactive protein level (184 mg/L). The patient had epigastric pain on palpation, but no signs of peritonitis. Repeated ultrasound of the abdomen did not show any new abnormalities.

A repeated abdominal CT revealed an increase in retroperitoneal free air and mesenteric fat infiltration, secondary to perforation of the duodenal diverticulum. The restriction from oral intake was prolonged, and total parenteral nutrition (TPV) was started. The patient was closely monitored with repeated physical examinations and blood analyses.

After an initial rise in c-reactive protein with a maximum of 253 mg/L, laboratory findings normalized within 7 days. At day 4, the fever resolved, and abdominal examination normalized. Two additional evaluating CT scans revealed reduction of retroperitoneal free air mesenteric fat infiltration. No abscesses were seen (Figure 1(b)). Eight days after admission, a regular diet was initiated. On hospital day 11, the patient was discharged with a regular diet. The patient was doing well at the follow-up visit three months after admission.

### 2.2. Case 2

A 63-year-old woman with diabetes mellitus type II, cholecystectomy, and appendectomy in her medical history presented with a sudden onset of pain localized in the right upper abdomen. The pain was associated with nausea and five episodes of vomiting. Her symptoms existed since the night prior to presentation. On examination, the patient was in mild distress with a body temperature of 38.0 degrees Celsius. Blood pressure and pulse rate were normal. The abdomen was not rigid, but there was direct tenderness on the right upper quadrant without evident muscle guarding.

**Figure 2:**
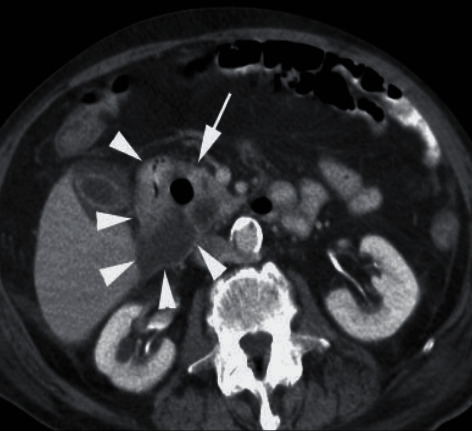
Duodenal diverticulitis with retroperitoneal perforation: (a) coronal CT scans at admission, (b) after 8 days of conservative treatment, and (c) after six weeks. The retroperitoneal air slowly resolves while the inflamed duodenal diverticulum (arrow) regains its normal aspect.

Blood analysis showed an evident leucocytosis (18.5∗10^3^ mm^3^ (4–10)) and normal c-reactive protein level (<5 mg/L (<5)). Subsequent CT imaging revealed a perforation of the duodenum and retroperitoneal gas in the right upper abdomen, most likely caused by a diverticulitis located at the proximal duodenum (Figure 2(a)). The patient was admitted, restricted from oral intake, and placed under close observation with serial abdominal exams and laboratory evaluation. Conservative treatment was started by broad spectrum antibiotics i.v. (metronidazole 3 dd 750 mg, cefuroxime 3 dd 750 mg, and gentamycin 1 dd 300 mg) together with pantoprazole, nasogastric decompression, and TPV. The patient was closely monitored with repeated physical examinations and blood analyses. On the first day of admission, CT imaging scan showed a deterioration of the situation with an increase of infection projecting from the site of perforation. Furthermore, blood analysis showed an increased C-reactive protein level (217 mg/L). However, the patient recovered clinically at the second day of admission and blood analyses normalized within 8 days. An additional CT scan at day 8 showed a strong reduction of surrounding retroperitoneal gas. No abscesses were seen (Figure 2(b)).

TPV was ceased at day 9. A regular diet was initiated, and patient was discharged at day 11 in good health. During follow-up, an additional CT scan was made (Figure 2(c)). Until now, she has never had any recurrent complaints concerning duodenal diverticula.

### 2.3. Case 3

**Figure 3:**
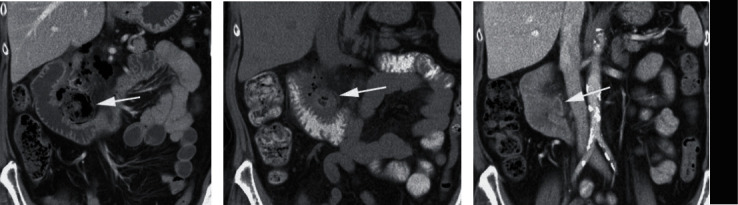
Duodenal diverticulitis. Axial CT scan shows inflamed diverticulum (arrow) medial to the duodenum (arrowheads). There was also free retroperitoneal air (not shown here).

A 74-year-old man, previously diagnosed with an adenocarcinoma of the right lung for which he was being treated by radiotherapy, presented with gradually increasing continuous pain in his right upper abdomen, radiating to the back. He was allergic to cefuroxime. The pain existed for five days. On physical examination, there was tenderness in the right upper abdomen with mild muscle guarding. He had a subfebrile temperature of 37.9 degrees Celsius. Blood pressure was 145/85 mmHg; pulse rate was 85 beats/min. Blood analysis revealed a mild leucocytosis (12.9∗10^3^ mm^3^ (4–10)) and an elevated c-reactive protein level (255 mg/L (<5). CT imaging revealed retroperitoneal air, most likely caused by perforation of a duodenal diverticulitis located at the bulbus duodeni (Figure 3). The patient was admitted, restricted from oral intake, and placed under close observation with serial abdominal exams and laboratory evaluation. During gastroduodenoscopy, which was undertaken to place a triple-lumen tube beyond the site of perforation, duodenitis near the bulbus was observed. Broad-spectrum antibiotics were started (metronidazole 3 dd 750 mg, amoxicillin 4 dd 1000 mg, and gentamycin 1 dd 320 mg). The patient received nutrition through the triple-lumen tube. During an admission of twenty days, the patient's temperature normalized and the patient recovered clinically. An additional CT scan at day 20 was consistent with the clinical findings; however, the site of perforation was still not entirely healed. Therefore, the patient was discharged with oral antibiotics (Augmentin 4 dd 625 mg). At the follow-up visit after four weeks, the patient was free of abdominal complaints. 18 months after admission, the patient died of pneumonia above progression of his lung cancer.

## 3. Discussion

Up to 22% of the healthy population is affected by duodenal diverticula [1]. They are asymptomatic until they develop complications, which occur in about 10% of patients with duodenal diverticula. Many possible complications have been reported. The most frequent are ulceration, inflammation, haemorrhage, fistula formation, pancreatitis, and common bile duct obstruction. Perforation is considered to be the rarest complication, but also the most threatening, with a mortality of up to 20% [1]. Diverticulitis is considered the most frequent cause of perforation. Additionally, other factors which may precede perforation include enterolithiasis, ulceration, increased intraluminal pressure, foreign bodies, trauma, gallstones, and iatrogenic causes such as feeding or suction tubes or endoscopy [1, 7, 14].

Diagnosis is difficult, because the clinical presentation is nonspecific. Most frequent findings are pain in the right upper abdomen or epigastrium, associated with nausea and vomiting. As perforation is frequently directed to the retroperitoneum, classical signs of peritonitis are not often present. Because of the lack of pathognomonic signs or symptoms, perforated duodenal diverticulitis is often clinically mistaken for acute cholecystitis, appendicitis, and perforated duodenal or gastric ulcer. In earlier decades, true diagnosis of perforated duodenal diverticulitis was made intraoperatively in most cases and it was rarely confirmed by radiographic studies. Up to 1992, Duarte et al. reported a correct preoperative diagnosis in only 13 out of 101 patients due to radiographic studies. In those series, fifty percent of the plain radiographs were normal, with 27 percent demonstrating gas retro- or paraduodenally. In only three cases, the diagnosis was confirmed by abdominal computed tomography. However, recent studies show more promising results from radiographic studies as a diagnostic tool preoperatively, with CT now being the diagnostic modality of choice. Usually, a thickened duodenal wall is seen, with mesenteric fat inflammation and an extraluminal collection of air and fluid. It is frequently possible to identify the diverticulum itself [5, 6]. Indeed, in our series, the diagnosis was confirmed in all cases by CT. However, one should be aware that CT findings may be misleading and raise the suspicion to more common diagnosis [3].

With only about 176 reports of perforations of duodenal diverticula described in literature, the optimal management of perforated duodenal diverticula remains uncertain [12]. Management is generally guided by the clinical condition of the patient and the preoperative diagnosis. Because of preoperative diagnostic uncertainty in previous decades, surgical treatment has been performed in most cases, and conservative management has rarely been described. Therefore, there is no general consensus in literature for an antibiotic regimen in the case of conservative treatment. In our cases, antibiotic regimens were composed according to local protocol at the time of treatment, and different regimens could be administered in different cases.

The type of surgery will depend on the clinical situation and intraoperative findings [15]. Postoperative complication rates are as high as 41% and include suture line leak, duodenal fistula, sepsis, and intraabdominal abscess formation [2, 16]. Most common surgical techniques described are diverticulectomy either by laparoscopy or laparotomy with single- or double-layer duodenal closure and subtotal gastrectomy followed by Billroth II reconstruction or a Roux-en-Y gastroenteroanastomosis [17]. Depending on the location of perforation, either partial or total duodenectomy is indicated. In 2018, di Saverio et al. described several cases in which a major duodenal perforation in D1 or D2 was treated by ampulla-preserving and pancreas-sparing duodenectomy by both laparotomy and laparoscopy, with a mortality of 20% [18]. When the perforation is located in D3 or D4, a total duodenectomy can be required. Successful treatment by pancreatoduodenectomy has been described [16]. Also, a pancreas-sparing complete duodenectomy has been attempted, but detachment and reimplantation of the duodenal ampulla seem technically more demanding and are associated with high postoperative leakage and complication rates [19, 20].

In recent years, several publications reported on treatment of perforated diverticulitis or a subsequent abscess by endoscopic therapy. Endoscopic nasogastric drainage or endoscopic transluminal water irrigation has been described as a feasible successful treatment for specific cases [21–24].

Patients with mild symptoms and no septic signs could benefit from nonoperative management, consisting of nasogastric decompression and broad-spectrum antibiotics. The last decade, reports of cases who were successfully treated conservatively are increasing. In these patients, the perforation has probably already sealed spontaneously or will do so within a short period of time.

Besides regular physical examination and blood analyses, it may be advised to repeat radiographic studies on a regular basis to evaluate whether continuing conservative treatment is safe. Before starting oral alimentation, radiographic studies with water-soluble contrast are advised. Formation of abscesses is likely, so percutaneous or endoscopic drainage must be easily accessible if conservative management is implemented. If a patient's condition worsens, conservative management must be abandoned in favour of surgery. Although conservative treatment is warranted in mild cases, sometimes, surgery cannot be avoided. In more severe cases or giant perforations, early recognition of need for surgical treatment remains essential for optimal treatment and source control. Consultation of a referral centre in an early stage is advised in more severe cases in case of lack of hepatobiliary expertise.

## 4. Conclusions

In conclusion, perforated duodenal diverticulitis is a very rare but serious complication with a difficult diagnosis. In the past decades, intraoperative diagnostic confirmation has been replaced by mostly preoperative diagnosis nowadays due to CT scan studies. Since very few cases with conservative treatment of this disease have been reported, this study contributes to the total amount of successful conservative treated cases described in literature.

In this era in which CT scan has become a commonly and frequently used preoperative diagnostic tool, conservative treatment should be considered in patients who are in generally good condition with no septic signs and symptoms. Close monitoring of the patient is necessary, so that conservative management can be replaced by surgery the moment the patient deteriorates.

## Data Availability

Data are withdrawn from physical and digital medical files. Data is enclosed in physical and digital patient files and therefore not accessible to readers.
